# Effectiveness of anger‐focused emotional management training in reducing aggression among nurses

**DOI:** 10.1002/nop2.1367

**Published:** 2022-09-16

**Authors:** Yuriko Tanabe, Takeshi Asami, Asuka Yoshimi, Kie Abe, Yusuke Saigusa, Maya Hayakawa, Junichi Fujita, Keiko Ide, Akira Suda, Akitoyo Hishimoto

**Affiliations:** ^1^ Department of Psychiatry Yokohama City University Graduate School of Medicine Yokohama Japan; ^2^ Department of Nursing Yokohama City University School of Medicine Yokohama Japan; ^3^ Department of Psychiatry Yokohama City University School of Medicine Yokohama Japan; ^4^ Tsukuba Psychosomatic Clinic Tsuchiura Japan; ^5^ Department of Biostatistics Yokohama City University School of Medicine Yokohama Japan; ^6^ Department of Child Psychiatry Yokohama City University Hospital Yokohama Japan

**Keywords:** aggression, anger management, inappropriate care, nurse, patient safety

## Abstract

**Aim:**

The aim of this study was to conduct a 5‐h training programme on anger‐focused emotional management for nurses and verify its effectiveness.

**Design:**

The study used a one‐group pretest–posttest design.

**Methods:**

Participants (*N* = 283) attended a programme comprising lectures and exercises. The Japanese version of the Buss–Perry Aggression Questionnaire was administered pre‐, post‐ and 3‐month posttraining. Regression analyses were used to assess the effects of the programme by gender.

**Results:**

For the total aggression score, the difference between the pre‐ and posttraining scores was −2.827 points and remained at −1.602 points 3‐month posttraining. Physical aggression scores decreased posttraining, but the scores increased after 3 months. There were statistically significant gender differences in hostility scores; pre‐training scores were slightly higher for men than for women and lower for men after 3 months. Total and physical aggression scores were higher for men than for women. The training programme decreased aggression, and the effect persisted after 3 months.

## 1 INTRODUCTION

The emotional management of human service workers is a common issue. In nurses, poor emotional management can negatively impact team dynamics, communication efficiency and task accountability, all of which can adversely affect patient care (Molina‐Praena et al., 2018; Rosenstein & Naylor, 2012). Poor emotional management may also lead to the deterioration of nurses' mental health (Han et al., 2015; Hu et al., 2014), causing staff burnout and turnover (Edward et al., 2017; Rosenthal et al., 2018). In recent research, emotion dysregulation was reported to be associated with aggression (Cohn et al., 2010). Moreover, individuals that have trouble managing their emotions have been found to be more probably to have abused their partner (Tager et al., 2010). In light of this, it is possible that nurses with poor emotional management may display aggressive behaviour towards patients, as well as their families and colleagues. Anger and other emotional management are required to reduce such aggression. Therefore, there is a growing demand for educational programmes to develop nurses' emotional competence (Prado‐Gascó et al., 2019).

## 2 BACKGROUND

Emotion is defined as a complex reaction pattern, involving experiential, behavioural and physiological elements (Novaco, 2007). The emotion of anger is very natural and intense when one is faced with frustrating or difficult situations (Dollard et al., 1939) and precedes aggressive behaviours more than any other negative emotion (Novaco, 2007). Anger also may be the most focused upon emotion in interpersonal work.

Nurses experience high stress due to a heavy workload and emotional labour. Research has shown that aggression from patients and their families is common in the medical setting (Shi et al., 2017). Particularly, nurses who provide direct care to patients are at high risk of being targets of violence (Ramacciati et al., 2018). Patient violence towards nurses could lead to increased aggression and violence by nurses towards patients (Laeeque et al., 2019). Nurses may also direct their aggression towards colleagues and subordinates. Such negative interactions can interfere with their ability to work (Bambi et al., 2018).

According to a review, a broad range of maladaptive styles of emotion regulation can increase aggressive behaviour. The influence of the under‐regulation of anger on aggression is particularly clear (Roberton et al., 2012). The aforementioned review showed that three skills (emotional awareness, emotional acceptance and proficiency in a variety of emotion regulation strategies) may lessen aggressive behaviour. Anger‐focused management interventions have also shown moderate effects for nurses (Willner et al., 2013).

Therefore, this study focused on the management of the main source of aggression, anger. Anger management was developed based upon psychoeducation and cognitive behavioural therapy, focusing on the management of anger that precedes aggression (Novaco, 1975, 1976). In a previous study conducted with nurses, a psychoeducational programme on anger management affected the psychological resilience and emotional state of nurses (Turan, 2021) and led to greater job satisfaction (Farahani & Ebadie Zare, 2018). It also led to improvements in levels of anger, job stress, psychological well‐being and heart rate (Yun & Yoo, 2021). These effects persisted after 4 weeks (Yun & Yoo, 2021). In Japan, anger management training for staff is not yet widely available; thus, there is a need to investigate the validity of anger management programmes for nurses in Japan.

In this study, we conducted a 5‐hr 1‐day training programme for the anger‐focused emotional management of nurses in order to reduce their levels of aggression. The current study focuses on the effects of anger management on aggression, as well as their persistence over 3 months.

## 3 THE STUDY

### 3.1 Design and procedure

The research question of this study was whether clinical nurses who attend an emotional education programme report reduced aggression levels. The programme evaluation in this study used a one‐group pre‐test‐posttest design and was conducted from June 2018 to March 2020.

### 3.2 Training programme overview

An aggression management training programme for healthcare providers is an intervention for deterring inappropriate expressions of aggression towards patients, preventing abuse, improving collaboration among staff, tackling interpersonal issues in the workplace and improving staff members' ability to self‐manage their emotions. The programme was devised to provide effective learning of the knowledge and skills necessary for healthcare providers. From the case studies collected in the preceding training, the teaching cases were created by identifying clinical situations that affect nurses' emotions. The training programme comprised a 5‐hr 1‐day session that involved group education with a combination of lectures and exercises. The content on anger management was based on a programme developed by the Japan Anger Management Association, a chapter of the National Anger Management Association, and was modified specifically for nurses. Assertive communication was incorporated into the training to address expressions of anger. The course covered the following topics: basic knowledge about emotions including anger, knowing one's anger tendencies, coping with impulses, stress coping, gaining awareness of the values that cause anger and assertive communication of anger expressions (Table 1). Each topic was covered through lectures, individual work and group share work to help participants realize their own issues and adopt techniques that suited them. This study was conducted by a single trainer (YT), and there was no difference in instruction for each group. Group work was conducted on the topic of the participants' own clinical experiences. It was structured so that, by sharing their own experiences with others, they could become aware of their own tendencies of thought and identify differences in their values. During group work, the trainer approached each group and gave them advice when needed. The number of participants in the education programme ranged from 30 to 60 per day, and each training group was kept small and consisted of no more than four persons for more effective learning. The education programme was organized and delivered by the author (YT).

**TABLE 1:** Overview of the anger‐focused emotional management education programme

Contents	Time (min)	Components of programme
Introduction	30	Explain the procedure for group work Introduction and rational of programme
Knowledge of emotions	60	Lecture on basic knowledge of emotions Issues related to emotion in clinical practice Group discussion
Know your anger tendency	60	Lecture on the individual characteristics of emotions Self‐insight into the characteristics of emotions
lunch break	60	
Control your impulsiveness	30	Lecture on skills related to anger management Group discussion on coping skills
Mental health management	30	Lecture on knowledge of stress management Group discussion on stress coping
Identify your beliefs	50	Knowledge of cognitive behavioural therapy Group discussion on clinical organizational culture
Communication training	30	Lecture on communication skills Assertive communication practice
Closing	10	Use of emotional management in clinical practice

*Note*: The time taken to complete the pre‐ and posttraining questionnaires was not included in the programme time.

### 3.3 Data collection

The training programme was organized by the prefectural professional associations of eight prefectures in Japan. We made an open call for participants from nurses who had taken the training programme, and a questionnaire survey was conducted with the permission of the training host organization. The regions where the training sessions were held were distributed in an unbiased manner. The sample size was calculated based on feasibility, size of the training programme. Before the training began, participants were informed that there would be three surveys—pre‐training, posttraining and 3 months after—and those who agreed participated in the surveys.

On the day of the training, the questionnaire surveys were conducted before and after the session. To evaluate the continuation of the training effect, a follow‐up survey was mailed 3 months later to those who had responded to the pre‐ and postsurveys on the day of the programme.

The questionnaire included items on gender, job title, age, years of clinical experience and the Japanese version of the Buss–Perry Aggression Questionnaire (BAQ), the most popular self‐report measure of aggression. An indicator of aggression was used to assess the impact of the programme, because the training programme was designed to improve inappropriate human care, abuse and harassment in clinical settings—behaviours that are associated with aggression (Bloom, 2019). The questionnaire was developed by Buss & Perry (1992), and the reliability and validity of the Japanese version have been verified (Ando et al., 1999). It comprises four subscales: anger, hostility, physical aggression and verbal aggression. The original scale consists of 24 questions across the four subscales, with possible scores ranging from 24–120 points. When translated into Japanese, two questions were excluded from scoring as irrelevant items. Participants rated items on a five‐point scale, ranging from 1 (*strongly disagree*) to 5 (*strongly agree*). Scores were added for each subscale after flipping the responses for inverted questions. The total score ranged from 22–110 points, with individual subscale scores of 5–25 (anger), 6–30 (hostility), 6–30 (physical aggression) and 5–25 (verbal aggression).

### 3.4 Participants

Participants in the study included qualified nurses working in hospitals who took part in the training programme. Before the training, we explained the purpose of the study to the participants and obtained their informed consent. To ensure accurate measurement of the effect of the training, those who had previously received training in anger management were excluded. The pre‐, post‐ and 3‐month posttraining surveys were all conducted anonymously. To enable accurate link pairing of the three pre–post questionnaires for each participant, the participants were assigned an arbitrary four‐digit identification number. After 3 months, we sent the questionnaire to each participant, who required to write down the four‐digit number that gave them at the start of the programme. Individuals for whom questionnaires could not be matched or who had numerous missing answers were excluded. The questionnaires were checked for the confirmation of consent to participate in the study, and qualifying questionnaires were included in the analysis. The final analysis included 283 participants who completed the surveys pre‐ and posttraining and 213 participants who completed the survey 3 months after the training.

### 3.5 Statistical analyses

All statistical analyses were performed using IBM SPSS Statistics version 26.0 (IBM Corp.). Descriptive statistics was used to analyse data. Regression analyses were performed using a linear mixed‐effect model to assess the differences of aggression scale scores at the three time points (pre‐, post‐ and 3‐month posttraining) and gender, with each scale score as a response variable pre‐ and 3‐month posttraining. The model including fixed effects and a random intercept for participants was examined for the interaction of time (three time points), gender (men/women), years of clinical experience, time × gender, time × age and time × years of experience. Akaike's information criterion was used for model selection.

### 3.6 Ethical considerations

This study was approved by the Ethics Review Committee. No information could identify an individual in the questionnaire, and the answers were anonymous. We provided participants with an explanation of the study and procedures and obtained their written informed consent.

## 4 RESULTS

Of the 283 participants, 87 (30.7%) were men and 196 (69.3%) were women; the sample had a higher percentage of men than that of the national nursing population. The mean age was 42.65 years (standard deviation [*SD*] = 9.55, range = 23–68). The mean years of clinical experience were 17.92 (*SD* = 10.02, range = 1–43). The job titles were general staff, 208 (73.5%), and manager, 75 (26.5%).

**FIGURE 1:**
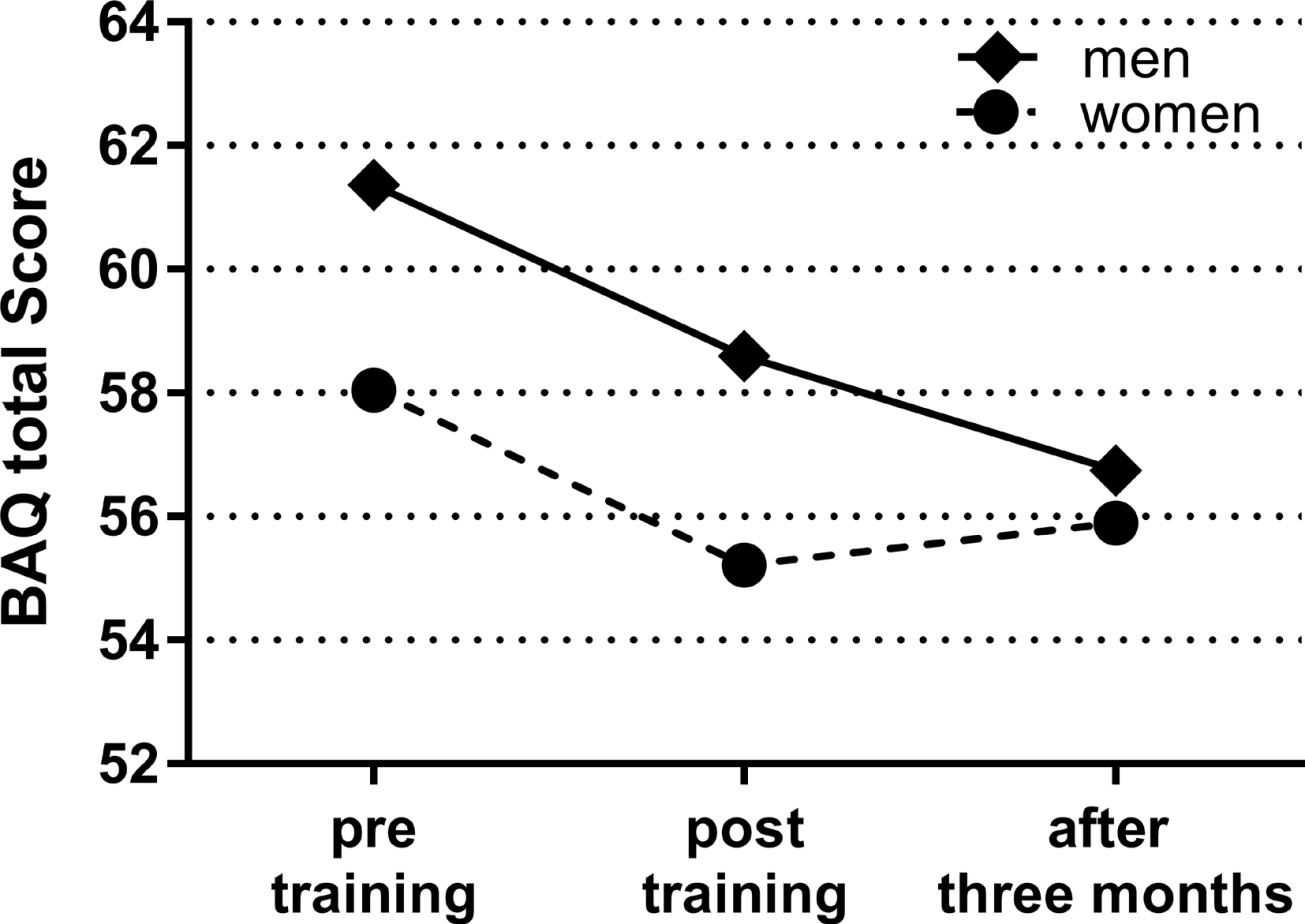
Comparison of total BAQ scores by gender. The average score of the measurement

The pre‐training aggression score was the baseline (Table 2). Table 3 shows the score estimates immediately after training and 3 months after the training as compared with the baseline. The difference between the pre‐ and posttraining total scores was −2.827 points, and 3‐month posttraining, the effect was −1.602 points. At all three time points, men's total scores were higher than those for women (*p* = .016). The analysis of interaction of the total score indicated that, after 3 months, women's scores increased slightly, while men's scores continued to decrease (*p* = .057) (Figure 1).

**TABLE 2:** Baseline BAQ scores (*N* = 283)

Item (score range)	Mean ± SD	Range	95% CI (lower, upper)
Total BAQ score (22–110)	59.07 ± 10.00	33–96	57.90, 60.24
Anger (5–25)	14.52 ± 3.66	5–23	14.09, 14.94
Hostility (6–30)	17.07 ± 3.95	8–30	16.61, 17.53
Physical aggression (6–30)	13.53 ± 4.05	6–29	13.06, 14.01
Verbal aggression (5–25)	13.95 ± 2.93	5–25	13.61, 14.29

Abbreviation: BAQ, Japanese version of the Buss–Perry Aggression Questionnaire.

**TABLE 3:** Comparison of BAQ scores pre‐ and posttraining and after 3 months (*N* = 283)

Parameter	Total BAQ score					Anger					Hostility					Physical aggression					Verbal aggression				
	95% CI					95% CI					95% CI					95% CI					95% CI				
	Estimate	Lower	Upper	*t*	*p*	Estimate	Lower	Upper	*t*	*p*	Estimate	Lower	Upper	*t*	*p*	Estimate	Lower	Upper	*t*	*p*	Estimate	Lower	Upper	*t*	*p*
Intercept	60.227	54.369	66.085	20.237	.000	14.417	12.279	16.556	13.270	.000	18.342	15.848	20.836	14.474	.000	13.223	10.847	15.599	10.953	.000	14.248	12.584	15.912	16.850	.000
Gender	3.019	0.559	5.479	2.413	.016*	0.414	−0.488	1.315	0.903	.367	0.157	−0.880	1.195	0.299	.765	2.084	1.049	3.119	3.956	.000**	0.365	−0.359	1.090	0.991	.322
Age	−0.047	−0.229	0.135	−0.506	.613	0.003	−0.064	0.069	0.087	.930	−0.046	−0.124	0.031	−1.176	.241	0.011	−0.062	0.085	0.306	.760	−0.015	−0.067	0.036	−0.580	.562
Years of experience	−0.005	−0.179	0.169	−0.055	.956	−0.009	−0.072	0.055	−0.266	.790	0.037	−0.038	0.111	0.973	.332	−0.046	−0.116	0.025	−1.274	.204	0.013	−0.036	0.063	0.533	.595
After 3 months	−1.602	−2.632	−.572	−3.055	.002**	−0.763	−1.149	−0.376	−3.880	.000**	−0.579	−0.987	−0.172	−1.792	.005**	−0.207	−0.723	0.309	−0.788	.431	−0.080	−0.442	0.281	−0.436	.663
Posttraining	−2.827	−3.781	−1.872	−5.821	.000**	−0.934	−1.291	−0.576	−5.128	.000**	−0.668	−1.046	−0.291	−3.481	.001**	−1.199	−1.678	−0.719	−4.916	.000**	−0.026	−0.361	0.310	−0.149	.881
Pre‐training	0					0					0					0					0				
After 3 months × Gender	−1.943	−3.944	0.058	−1.908	.057	−0.383	−1.133	0.367	−1.003	.317	−0.801	−1.593	−0.009	−1.988	.047*	−0.780	−1.782	0.221	−1.531	.126	−0.001	−0.702	0.699	−0.004	.997
Posttraining × Gender	0.045	−1.676	1.766	0.051	.959	0.014	−0.631	0.659	0.043	.966	−0.056	−0.736	0.625	−0.161	.872	0.027	−0.838	0.891	0.060	.952	0.060	−0.545	0.665	0.195	.846
Pre‐training × Gender	0					0					0					0					0				

*Note*: All other interaction terms were not selected based on an AIC that was not significant.

Abbreviations: BAQ, Japanese version of the Buss–Perry Aggression Questionnaire; *p*, probability value.

* *p* < .05.

** *p* < .01.

In the analysis of subscales, the anger and hostility scores decreased significantly in the posttraining, and the effect remained 3 months after training in both subscale scores, as well as in the total score.

**FIGURE 2:**
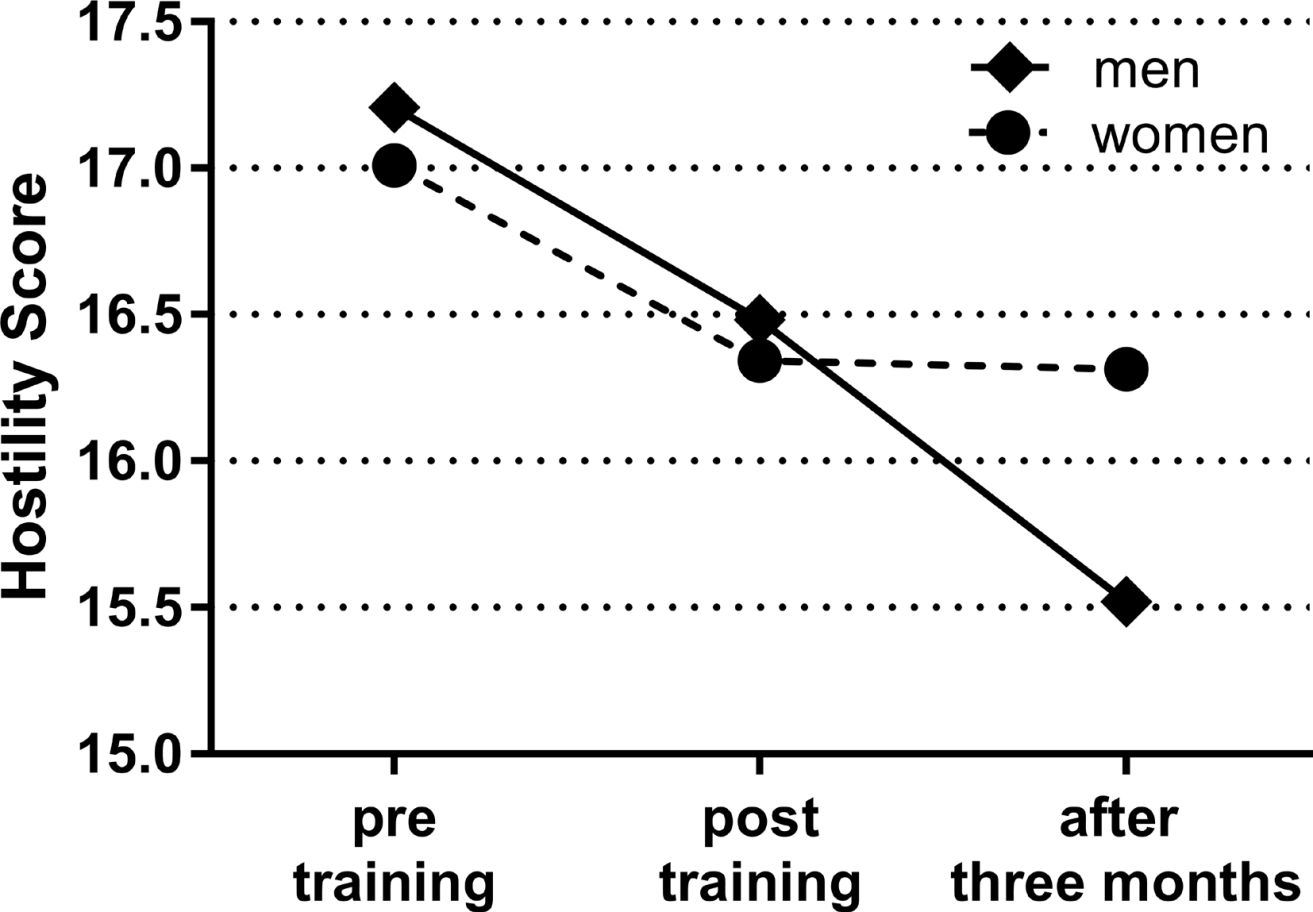
Comparison of hostility scores by gender. The average score of the measurement

The hostility score at posttraining was significantly smaller than that at pre‐training (estimated coefficient: –0.668; 95% CI: −1.046 to –0.293; *p* = .001) and after 3 months (−0.579; −0.987 to –0.172; .005), with a statistically significant difference between men and women at pre‐training and after 3 months (*p* = .047). Pre‐training scores were slightly higher for men than for women and lower for men 3 months after the training (Figure 2; Table 3).

The physical aggression score was −1.199 points (95% CI: −1.678 to –0.719; *p* < .001) posttraining compared with pre‐training, but −0.207 points (95% CI: −0.723 to –0.309; p < .001) after 3 months, and there were no statistically significant differences between the scores at the pre‐training or after 3 months. Men's physical aggression scores were higher than women's at all time points (*p* < .001).

There was no change in verbal aggression scores at posttraining or after 3 months compared with the pre‐training score.

The Cronbach's alpha values for the BAQ were .893 (anger), .902 (hostility), .823 (physical aggression), .830 (verbal aggression) and .888 (total). Finally, there were no statistically significant differences in the other interaction terms (time × age, time × years of experience).

## 5 DISCUSSION

### 5.1 Changes in aggression scores due to implementation of educational programs

The main finding of the current study was that total aggression scores decreased significantly immediately after the training, and the effect remained for 3 months. Among the subscales, the anger and hostility scores decreased significantly in posttraining compared with pre‐training and were maintained until 3 months after the training. The results show that this educational programme can reduce nurses' emotional aggression. The results also showed that the effects persisted after the training. Reducing nurses' aggression towards patients and staff may have the effect of reducing abuse of patients. It is thus worthwhile to implement this training programme for nurses.

Previous studies on anger management programmes for nurses reported improved levels of anger (Yun & Yoo, 2021) and aggression (Farahani & Ebadie Zare, 2018), as well as psychological resilience, emotional state (Turan, 2021), job satisfaction, psychological well‐being and heart rate (Farahani & Ebadie Zare, 2018; Yun & Yoo, 2021). Our results are in line with these studies. In terms of the duration of the effectiveness, two studies reported continued effectiveness at 4‐week postprogramme (Turan, 2021; Yun & Yoo, 2021). Our results showed that our programme was still effective at 3‐month postprogramme; however, more research may be needed to confirm for how long the effect of each programme lasts.

Training using a cognitive‐behavioural model of anger has been validated as providing participants with the ability to understand and respond to patients' anger (Gerhart et al., 2017), and the results of the present study supported reduced levels of aggression posttraining, suggesting that the effectiveness of the education programme was validated in terms of reduced aggression among nurses.

The physical aggression scores decreased immediately after implementation of the training programme but increased after 3 months, and no statistically significant effect of the emotional management education programme was observed. It is presumed that learning about emotions motivates nurses to improve their cognition and expression of aggression, but when they return to their daily clinical practice, they return to their original cognitive and behavioural patterns. For nurses to improve their anger control when dealing with patients, their emotional management needs to improve, manifest itself as behaviour and remain effective over time, especially with regard to physical aggression. Anger management is a behaviour based on previous beliefs, making it easy to gradually return to the original pattern. Repeated stimulation is necessary to maintain the effect (Kwansnicka et al., 2016).

Studies of training in institutions have demonstrated the following: an 8‐week anger management programme had an impact on psychological resilience and nurses' emotional states (Turan, 2021); multiple sessions of stress management improved the quality of nursing care (Pahlavanzadeh et al., 2016); continuing education interventions result in positive nurse‐related outcomes such as increased knowledge, confidence and skills, or improved attitudes (Hartley et al., 2019) and lead to broader organizational improvements (Bryant & Posey, 2019); and structured training programs with simulation scenarios, reflective exercises and role‐playing could be useful to facilitate nurse learning (Labrague & McEnroe‐Petitte, 2017). Our programme takes only 5 hr of 1 day, and yet it impacted anger and hostility levels. It may be efficient compared with other programmes; however, it did not affect physical aggression after 3 months or verbal aggression at any point. The development of training that incorporates continuing education and repetitive learning about anger regulation is necessary. It may also be necessary to combine such programmes with aggressive management training (Paterson et al., 1992; Stubbs et al., 2009).

To prevent inappropriate care, behaviours such as physical and verbal aggression must be reduced. Verbal aggression is more common than physical aggression, with verbal aggression affecting the health of staff more strongly than external violence. It has been reported that verbal aggression is also considered the most common type of workplace aggression (Pien et al., 2019). Physical aggression and verbal aggression are risk factors in nursing practice due to concerns about harming others. Appropriate anger expression could result in enhanced interpersonal competency via an increase in self‐efficacy (Jun, 2016). Most healthcare organizations require training programmes to reduce verbal aggression. In this study, scores for physical aggression decreased, while scores for verbal aggression did not change. Since this programme is proportional to learning anger emotions, including cognition, and communication training is only one part of the programme, it may not have yielded changes in scores. Previous research has shown that verbal aggression is positively correlated with physical aggression (Fino et al., 2019), so it is possible that verbal aggression may change in the long term. Assertiveness was incorporated as an expression of anger in the training. It has been shown to have a fixed effect with prolonged training (Yoshinaga et al., 2018). It has been reported that the cultural background of nursing in Japan includes understaffing and difficulty in taking or requesting days off, which is associated with workplace bullying (Yokoyama et al., 2016). Since assertiveness comprises skills for expressing rather than suppressing emotions, it may lead to improvements in how nurses express themselves, although this effect was absent in the verbal aggression scores.

### 5.2 Gender differences in aggression scores

Previous studies using the BAQ in Japan had shown men to be more physically aggressive than women (Ando et al., 1999; Kanchika et al., 2015). The physical aggression scores in this study were consistent with those of previous studies. With respect to gender differences in aggressive behaviour, depending on genetic determinants, men have been reported to use more physical aggression and women more indirect aggression (Björkqvist, 2018). Physical aggression has been reported to be lower for those with higher skills in intervening with patient aggression (Shimosato & Kinoshita, 2018). Gender differences in nurses' perception of patient aggression have been identified (Lickiewicz et al., 2020), and this study also found gender differences in nurses' own aggression.

Hostility scores did not differ by gender at pre‐ and posttraining, but there was a statistically significant difference in scores recorded 3 months after the training, with no change in scores for women but a decrease in scores for men. It has been reported that hostility does not differ by gender (Gerevich et al., 2007), and the current results, pre‐ and posttraining, were consistent with this finding. Furthermore, it is a new finding that there was a statistically significant difference in gender scores 3 months after training in the present study.

Hostility is a cognitive factor that implies a negative attitude towards others, wherein an individual believes that others are malevolent and, as a result, treats them unfairly (Buss & Perry, 1992). Repression and unhealthy expressions of anger can have negative effects on mental health, resulting in depression (Cha & Sok, 2014). Future research is required to investigate the relationship between hostility and other mental health measures to explore the influencing factors.

Surveys of nursing students have revealed several gender differences in academic, clinical, psychological, nursing professional identity and health concepts (Chan et al., 2014). This study suggests that there may be gender differences in training outcomes. Lickiewicz et al. (2020) reported the violence management training in medical school students was effective only for women. They stated that this difference may stem from gender differences in how aggression is perceived, as when compared to women, men tend to perceive aggression as less destructive (Bilgin et al., 2016). Immediately following the training, our programme was effective for both men and women; however, at 3‐month posttraining, the effect had diminished for women more than for men. Female nurses may face with the aggression from patients, their family and colleagues, and as a result, the effective of the programme may have diminished. Thus, female nurses may need additional trainings at shorter intervals than their male colleagues.

### 5.3 Limitations

This study was a single‐arm trial without a control group; thus, our results may be statistically significant due to the Hawthorne effect. Moreover, we did not eliminate the effect of confounding factors (e.g. cognitive‐behavioural techniques, group work or assertion). Future trials should be conducted with a control group to verify the effect of the programme. This study was conducted by a single trainer, and there was no difference in instruction by the trainer. By contrast, in group work, the trainer frequently approached each group and gave advice, but never looked at the group as a whole, which may have reduced the effectiveness of the programme. New findings on the long‐term effects of the training programme were obtained, but the progress beyond 3 months has not been confirmed. Improvements in survey methods are needed to identify long‐term changes in nurses' emotional management. Further research is warranted.

This study used the Japanese version of the BAQ, which was modified when it was translated to Japanese, resulting in different scores and, thus, limiting the comparability of international scores. Although this study showed gender differences in aggression, it did not capture gender characteristics in an unbiased manner, as the participants of this study were nurses, and the nursing workforce is predominantly composed of women. The type and size of the hospital to which the participants belonged were not surveyed. Therefore, further surveys should be conducted to correct for differing gender proportions and departments. This study used a method of analysis that compensates for dropouts, but some of the subjects who dropped out of the training programme may not have demonstrated an educational effect. Additionally, the use of only self‐reported measures of aggression limits its objectivity. Although the effects in clinical practice have not been confirmed, there are reports of a relationship between aggression and behaviours, such as patient abuse and workplace bullying (Bloom, 2019), which suggests that the results of this study may be effective. Therefore, verification of the effects in clinical practice is required in the future.

## 6 CONCLUSION

In this study, the Japanese version of the BAQ was used to measure the effectiveness of anger‐focused emotional management training on reducing aggression. The total score of the aggression decreased when the nurses underwent an anger‐focused emotional management training programme, and the effect persisted after 3 months. Physical aggression scores decreased posttraining, but the scores increased after 3 months. There was no statistically significant difference in verbal aggression between the three time points. The study concludes that effective emotional education for nurses requires repetitive training.

Emotional management is required for nurses to continue to provide high‐quality nursing care (Ebrahimi et al., 2016). Therefore, it should implement an integrated training programme for nurses and provide continuous emotional support.

## AUTHOR CONTRIBUTIONS

YT and TA: Study conceptualization and design. YT: Data collection, dataset processing, data analysis performance and manuscript writing. YT, TA, AY, KA and YS: Data analysis. MA, JF, KI, AS and AH: Manuscript revision. All authors have agreed with and approved the final manuscript.

## FUNDING INFORMATION

This work was supported by JSPS KAKENHI (17 K12164) Grant‐in‐Aid for Scientific Research(C).

## CONFLICT OF INTEREST

The authors declare no conflict of interest.

## ETHICS STATEMENT

This study was approved by the Ethics Review Committee of Yokohama City University (#A180500006).

## Data Availability

The data that support the findings of this study are available from the corresponding author upon reasonable request.
